# Surface electroencephalographic neurofeedback improves sustained attention in ADHD: a meta-analysis of randomized controlled trials

**DOI:** 10.1186/s13034-022-00543-1

**Published:** 2022-12-19

**Authors:** Hsien‐Jane Chiu, Cheuk-Kwan Sun, Hsin-Yi Fan, Ruu‐Fen Tzang, Ming-Yu Wang, Ying-Chih Cheng, Yu-Shian Cheng, Pin-Yang Yeh, Weilun Chung

**Affiliations:** 1 Taoyuan Psychiatric Center, Ministry of Health and Welfare, Taoyuan, Taiwan; 2 Institute of Hospital and Health Care Administration, National Yang Ming Chiao Tung University, Taipei, Taiwan; 3 Department of Emergency Medicine, E-Da Hospital, I-Shou University, Kaohsiung, Taiwan; 4 School of Medicine for International Students, College of Medicine, I-Shou University, Kaohsiung, Taiwan; 5 Department of Psychiatry, Tsyr-Huey Mental Hospital, Kaohsiung Jen-Ai’s Home, No. 509, Fengping 1St Rd., Daliao Dist, Kaohsiung, Taiwan; 6 Department of Psychiatry, Mackay Memorial Hospital, Taipei, Taiwan; 7 Department of Psychiatry, China Medical University Hsinchu Hospital, China Medical University, Hsinchu, Taiwan; 8 Department of Health Services Administration, China Medical University, Taichung, Taiwan; 9 Institute of Epidemiology and Preventive Medicine, College of Public Health, National Taiwan University, Taipei, Taiwan; 10 Research Center of Big Data and Meta-Analysis, Wan Fang Hospital, Taipei Medical University, Taipei, Taiwan; 11 Department of Psychology, College of Medical and Health Science, Asia University, 500, Lioufeng Rd., Wufeng, Taichung, 41354 Taiwan

**Keywords:** Biofeedback, Neurocognitive tests, Attentional performance, Informant bias

## Abstract

**Background:**

The efficacy of surface electroencephalographic neurofeedback (EEG-NF) for improving attentional performance assessed by laboratory measures in patients with attention-deficit/hyperactivity disorder (ADHD) remains unclear.

**Methods:**

Following the PRISMA guidelines, the PubMed, Embase, ClinicalKey, Cochrane CENTRAL, ScienceDirect, Web of Science, and ClinicalTrials.gov databases were systematically searched for randomized controlled trials on the efficacy of surface EEG-NF against ADHD focusing on attentional performance evaluated by laboratory measures from inception to January 2022.

**Results:**

Fourteen eligible studies were analyzed. Of the 718 participants involved, 429 diagnosed with ADHD received EEG-NF treatment. Significant improvement in attentional performance in ADHD subjects receiving EEG-NF was noted compared to their comparators (*p* < 0.01). Besides, there was a significant EEG-NF-associated beneficial effect on sustained attention (Hedges’ *g* = 0.32, *p* < 0.01), whereas the impact on selective attention (*p* = 0.57) and working memory (*p* = 0.59) was limited. Moreover, protocol including beta wave enhancement was superior to that only focusing on reducing theta/beta ratio or modulation of slow cortical potential. Subgroup analyses showed that three sessions per week of EEG-NF produced the best effect, while the efficacy of surface EEG-NF was much poorer (Hedges’ *g* = 0.05) when only studies that blinded their participants from knowledge of treatment allocation were included. No significant difference was noted in the improvement of attentional performance 6–12 months after EEG-NF intervention (n = 3, *p* = 0.42).

**Conclusions:**

Our results demonstrated the satisfactory effectiveness of surface EEG-NF for improving sustained attention, especially when beta wave enhancement was included, despite its failure to sustain a long-term effect. Further large-scale trials are warranted to support our findings.

**Supplementary Information:**

The online version contains supplementary material available at 10.1186/s13034-022-00543-1.

## Background

Attention-deficit/hyperactivity disorder (ADHD), which is characterized by symptoms of inattention and/or hyperactivity/impulsivity, is one of the most common neurodevelopmental disorders in children and adolescents [1]. Despite the reported efficacy of pharmacological interventions in a number of meta-analyses and reviews [2], parents of children with ADHD still frequently seek alternative treatments due to concerns about medication-related side effects [3]. Electroencephalographic neurofeedback (EEG-NF), which has been shown to be a promising alternative therapeutic option for symptoms of ADHD in previous randomized controlled trials (RCTs) and meta-analyses [4, 5], is a kind of biofeedback involving self-regulation of brain activity by providing an audio/visual feedback signal in response to the measured brain waves [6].

However, although previous evidence seemed to support the therapeutic effectiveness of EEG-NF for the symptoms of ADHD [6, 7], the treatment effect of EEG-NF was mostly evaluated with behavioral rating scales and only a few RCTs provided laboratory measures of attentional performance [6, 7]. While the former is rated by most proximal (i.e., parents) and/or possibly blind (e.g., teachers) evaluators, the latter involves computerized or paper neurocognitive tests as an objective assessment of the patient’s clinical condition [8]. Based on behavioral rating scales, the majority of previous meta-analytical studies [5, 7, 9–13] demonstrated positive treatment effects from the most-proximal evaluators, while the results from possibly blinded evaluators were inconsistent [5, 7, 9–13]. In contrast, an objective or computerized performance test may be less susceptible to informant bias [14] despite the lack of tangible evidence to support this proposal. In addition, instead of being a simple definition, the concept of attention is too complex [15] to be thoroughly evaluated with behavioral rating scales that could not provide specific information about the different aspects of attentional functions.

With regard to the treatment protocols of EEG-NF for ADHD, there are several strategies that target different patterns of brain waves or their combinations [e.g., theta/beta (TB)] [6, 16]. The TB protocol aiming at controlling hyperactivity and enhancing concentration [17] is one of the most popular options for patients with ADHD [6]. The slow cortical potential (SCP) protocol, which involves SCP modulation, has also been shown to improve the clinical symptoms of ADHD [18]. On the other hand, a recent study reported that frontal beta activity may be a better training target of EEG-NF compared to the TB protocol in ADHD patients with a longer reaction time [19]. Nevertheless, the treatment efficacy of the TB protocol in comparison with that of other therapeutic strategies remains unclear.

Notwithstanding the non-invasiveness of EEG-NF, its efficacy against the symptoms of ADHD has been controversial because of inconclusive evidence attributable to possible evaluator bias [20]. Some studies using a double-blind design failed to show superior effectiveness of EEG-NF compared to that of sham controls [21, 22]. There are also criticisms against its cost-effectiveness, time-consuming nature, and lack of long-lasting benefits [6]. Therefore, focusing on studies that used laboratory measures for theoretically more objective assessment of improvements in different components of attentional performance from surface EEG-NF, the present meta-analysis aimed at elucidating its therapeutic benefits in patients with ADHD. Furthermore, we also investigated the impacts of the intensity of treatment (i.e., number of sessions per week), quality of blinding, and different treatment protocols (i.e., beta vs. TB) on the therapeutic outcome in an attempt to provide the latest evidence for guiding clinical practice.

## Methods

### Study eligibility and definitions

For our literature search, we used different keywords, namely “neurofeedback” AND “attention or attention-deficit/hyperactivity disorder or ADHD” for identifying clinical trials (Additional file 1: Table S1). The criteria for study eligibility were: (1) random assignment of participants diagnosed as having ADHD to different treatment groups, (2) a comparison between subjects receiving EEG-NF and their comparators who were subjected to active or inactive treatments [e.g., waitlist or treatment as usual (TAU)])] other than pharmacological interventions, and (3) inclusion of attentional performance as an outcome measure of attention tests or cognitive tasks.

### Electronic searches

In accordance with the Preferred Reporting Items for Systematic Reviews and Meta-Analyses (PRISMA) guidelines [23, 24], we systematically searched the PubMed, Embase, ClinicalKey, Cochrane (CENTRAL), ScienceDirect, Web of Science, and ClinicalTrials.gov databases for English articles from inception to January 2022. The current study was registered with the international prospective register of meta-analysis (PROSPERO CRD 42021247674). The PRISMA checklist of the current meta-analysis is shown in Additional file 2: Table S2.

### Data extraction and management

Two authors (Cheng YS and Yeh PY) completed the title and abstract screening. Besides, full-text screening was independently conducted by Cheng YS and Yeh PY. Kappa statistics was calculated to assess inter-rater reliability [25]. Any disagreements were resolved through discussion between the two authors until consensus was reached. In case of missing information, we tried to electronically contact the corresponding authors for the original data. On encountering duplicated data, the article with the largest sample size or the latest information was chosen for analysis.

The validity of the eligible studies was assessed with the six criteria of the risk of bias assessment tool developed by the Cochrane Collaboration to target possible sources of bias [26], including random sequence generation, concealment of allocation to conditions, performance bias, detection bias, attrition bias, and reporting bias.

### Data synthesis and sensitivity analysis

Improvement in attentional performance was quantitatively expressed as effect size (ES) based on Hedges’ *g*. We used the computer program “Comprehensive Meta-Analysis version for Windows (CMA, version 3.3.070)” to calculate ESs, which were assigned a positive sign to indicate an improvement in attentional performance in subjects receiving EEG-NF. The outcomes of different assessment tools for testing attentional performance were categorized into three domains: (1) sustained attention, (2) selective attention, and (3) working memory. If a study provided data on only one domain, the data (merged when more than one set of data from different assessment tools) were used for the analysis of overall attentional performance. On the other hand, if a study offered results of more than one domain (e.g., integrated visual auditory continuous performance test for sustained attention and Stroop word and color test for selective attention), the results were standardized and averaged to produce a single ES. Regarding the evaluation of differences in magnitude of attention improvement, ESs of 0.8, 0.5, and 0.2 were interpreted as large, moderate, and small, respectively [27]. Besides, to tackle the problem of a diminished statistical power due to a small sample size commonly observed in studies involving psychological treatments, we used a random-effects model to estimate the ESs in this meta-analysis by assuming identical true ESs in all studies [28, 29]. This model allows the adjustment of sample size bias by offering an average distribution of effects across the included studies [29] so that the weights given to the studies may be more similar to each other [28].

In the current meta-analysis, we also performed subgroup analyses based on a random-effects model [28] to identify potential factors that may influence the observed therapeutic effectiveness of EEG-NF by categorizing the included studies according to: (1) Different treatment intensities defined as the number of NF sessions per week, (2) With or without blinding of treatment allocation to their participants (e.g. a sham control) based on the description of individual studies, and (3) Different EEG-NF protocols (e.g., TB or SCP protocols). Mixed-effects meta-regression was used to investigate the impact of continuous variables [e.g., intelligent quotient (IQ)] on the therapeutic effect of EEG-NF on attentional performance among patients with ADHD.. We also performed *Q* statistics and used the corresponding *p* values to assess the heterogeneity of ESs.

With respect to the evaluation of publication bias, funnel plots were inspected when there were fewer than 10 datasets [30], while Egger’s tests were performed if there were 10 or more datasets [31]. On encountering funnel plot asymmetry, potentially missing studies were imputed by using the Duval and Tweedie’s trim and fill method [32]. Sensitivity test was conducted with the leave-one-out approach through removing one study each time and repeating the step to estimate the effect of each study on the overall ES [30].

## Results

### Study characteristics

**Fig. 1:**
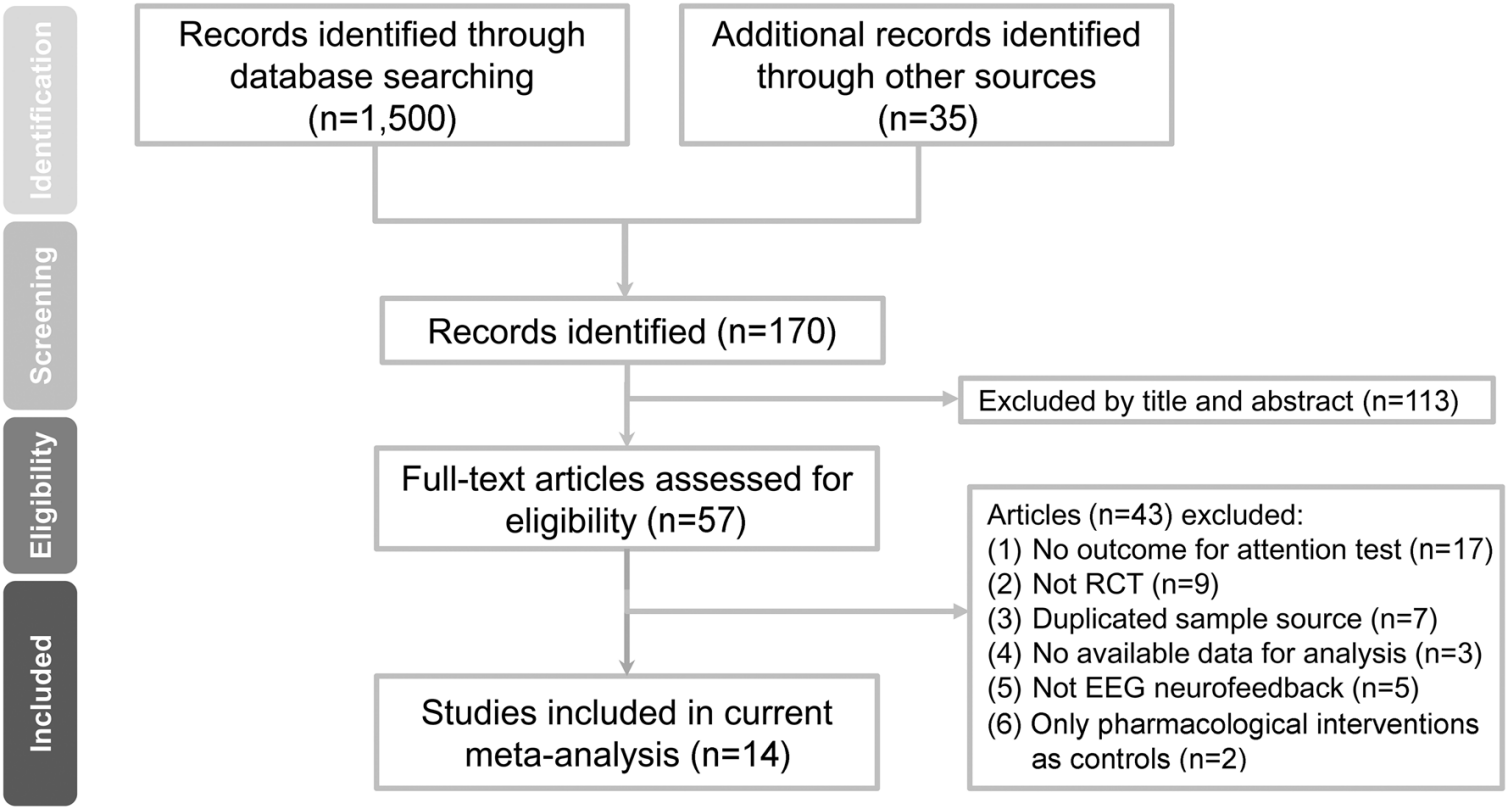
PRISMA diagram of identifying eligible studies

**Fig. 2:**
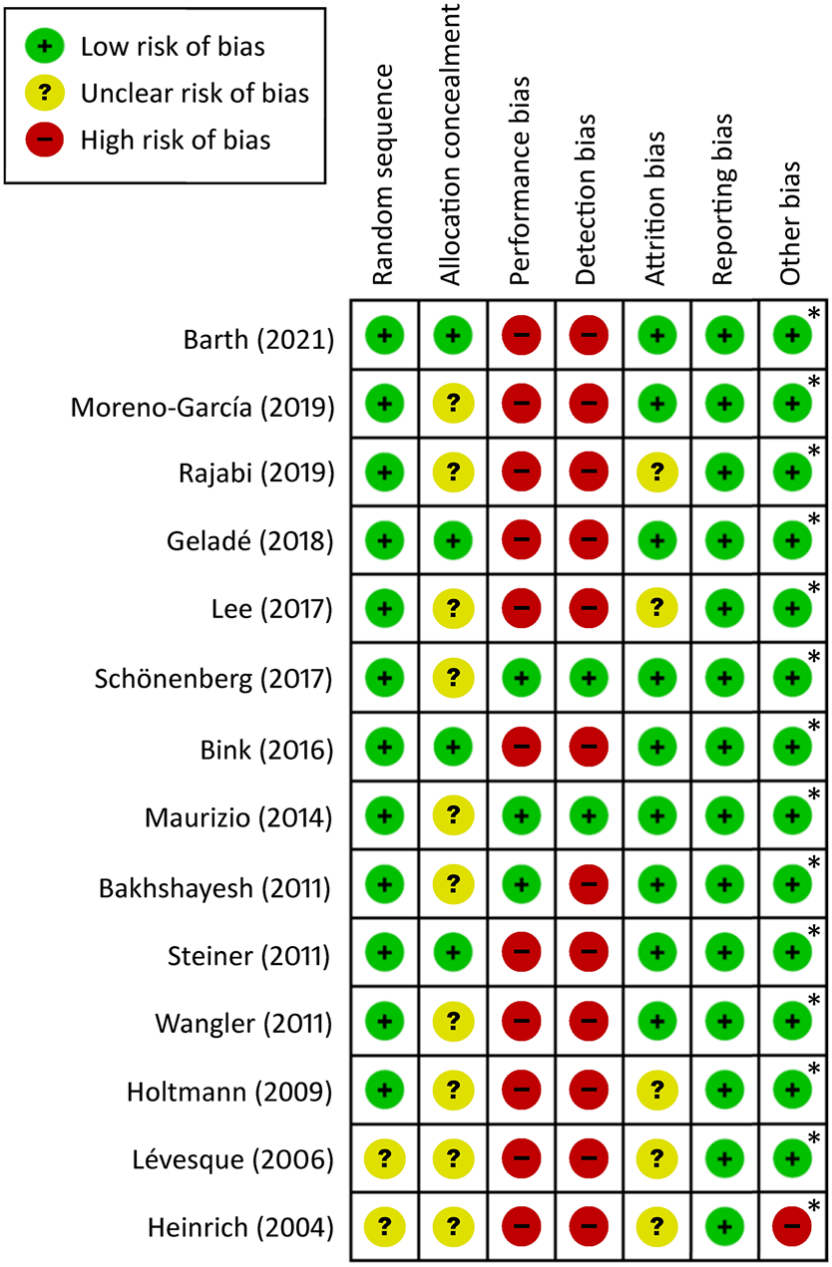
Risks of bias of the included studies. (Asterisk) both authors and studies received no financial support from pharmaceutical companies

Figure 1 summarizes the process of identifying eligible studies for the current meta-analysis. Of the 58 full-text articles assessed for eligibility, 44 were excluded for failing to meet the inclusion criteria. The reasons for exclusion are detailed in Additional file 3: Table S3. Finally, fourteen articles using the randomized controlled trial (RCT) design were selected for the current study [22, 33–45] (Table 1). The Kappa index of agreement was 1.0. The eligible studies and their risk of bias are shown in Fig. 2.

**Table 1:** Summary of characteristics of studies in the current meta-analysis

Author (year)	Criteria	Comparison		Session	N	Duration (weeks)	Outcome	Subtype			IQ	Stimulants (%)		Age (years)	Female (%)		Country
Barth (2021)	1. WURS-K ≥ 30 2. ADHS-SB ≥ 18	EEG-NF (SCP)		30	26	26.64	1. D2 Test-accuracy + concentration + speed 2. Go/No Go task – RT + omission + errors	NA			108.32	23.9		33.63 (18–56)	60.9		Germany
		EMG-BF			20												
Moreno-García (2019)	90th percentile ADHD-RS-IV (teacher)	EEG-NF (TB)		40	19	20	IVA CPT – Full Scale	Inattention (50%) Combined (31.58%) H/I (18.42%)			100.15 (KBIT)	0		8.66 (7–14)	23.5		Spain
		Behavioral therapy			19												
Rajabi (2019)*	DSM-5	EEG-NF (TB + beta training)		30	16	12	IVA CPT – Full Scale	Inattention (15.63%) Combined (59.38%) H/I (24.99%)			107.2	NA		10.13 (6–12)	0		Iran
		Waitlist			16												
Geladé (2018)	DSM-IV-TR	EEG-NF (TB)		30	33	12	1. Auditory oddball task – RT + CV 2. SST – RT + omission 3. VSWM- backward	NA			99.94	0		9.68 (7–13)	25		The Netherlands
		Physical activity			31												
Lee (2017)*	DSM-IV-TR	EEG-NF (TB + beta training) and Medication		20	18	10	1. ADS- inattention 2. Korean WISC-III – freedom from distractibility	Inattention (41.67%) Combined (44.44%) H/I (13.89%)			100.39	100		8.75	25		Korea
		Medications			18												
Schönenberg (2017)	DSM-IV-TR	EEG-NF (TB)		30	37	15	1. INKA 2. CPT – RT 3. Stroop word and color test	NA			NA	13.3		37.8 (18–60)	44		Germany
		Sham			38												
Bink (2016)	DSM-IV-TR	EEG-NF(TB)		37	39	25	1. D2 Test – total score 2. Digit span- backward 3. Stroop word and color test	NA			NA	48		15.95 (12–24)	0		The Netherlands
		TAU			19												
Maurizio (2014)	DSM-IV	EEG-NF (TB)		36	13	12	1. D2 Test – total score 2. KiTAP- RT + errors + hits + omission 3. TAP- RT	Inattention (0%) Combined (100%)			114.5 (German WISC)	8		10.31 (8.5–13.5)	12		Germany
		EMG-BF			12												
Bakhshayesh (2011)	ICD-10	EEG-NF (TB)		30	18	15	CPT – total score + omission + RT	Inattention (82.9%)			NA	20		9.34 (6–14)	25.7		Germany
		EMG biofeedback			17												
Steiner (2011)	By physician	EEG-NF (TB)		23.4	6	16	IVA CPT – attention quotient	NA			NA	60		12.4 (11–14)	47.8		USA
		SCF			10												
Wangler (2011)	DSM-IV-TR	EEG-NF (TB)		36	59	NA	ANT – alerting + conflict + orienting + hit + RT	Inattention (29.8%) Combined (70.2%)			105.5	7.4		9.68 (8–12)	18.1		Germany
		Attention skills training			35												
Holtmann (2009)	ICD-10 F90.0, F90.1, F98.8	EEG-NF (TB)		20	20	NA	SST-inattention	Inattention (58.8%) H/I (35.3%)			NA	79.4		10.3 (7–12)	8.8		Germany
		Computer-aided attention training			14												
Lévesque (2006)	DSM-IV	EEG-NF (TB)		40	15	13.5	1. IVA CPT 2. Stroop task- inference	NA			NA	0		10.2 (8–12)	20		Canada
		Control			5												
Heinrich (2004)	DSM-IV	EEG-NF (SCP)		25	13	3	CPT – Hits	Inattention (27.27%) Combined (72.73%)			112.9	45.45		10.81 (7–13)	4.5		Germany
		Waitlist			9												
Follow-up																	
Geladé (2017)]	DSM-IV-TR	EEG-NF (TB)	30		33	24	1. Auditory oddball task – RT + CV 2. SST – RT + omission 3. VSWM- backward		NA	99.94			0	9.68 (7–13)		25	The Netherlands
		Physical activity			31												
Schönenberg (2017)	DSM-IV-TR	EEG-NF (TB)	30		37	24	1. INKA 2. CPT – RT 3. Stroop word and color test		NA	NA			13.3	37.8 (18–60)		44	Germany
		Sham			38												
Bink (2016)	DSM-IV-TR	EEG-NF (TB)	37		39	52	1. D2 Test – total score 2. Digit span- backward 3. Stroop word and color test		NA	NA			48	15.95 (12–24)		0	The Netherlands
		TAU			19												

*ADHD* attention deficit and hyperactivity disorders, *ADHS-SB* German version of the ADHD self-rating scale, *ADHD-RS-IV* ADHD Rating Scale-IV, *ADS* ADHD diagnostic system, *ANT* Attention Network Test, *CPT* continuous performance task, *CV* coefficient of variation, *D2 test* test of attention, *DSM-IV* diagnostic and statistical manual of mental disorders, fourth edition, *DSM-IV-TR* diagnostic and statistical manual of mental disorders, fourth edition, text revision, *DSM-5* diagnostic and statistical manual of mental disorders, fifth edition, *EEG-NF* electroencephalographic biofeedback, *EMG-BF* electromyographic biofeedback, *KiTAP* test for attentional performance for children, *H/I* hyperactivity/impulsivity, *ICD-10* International Classification of Diseases, 10th Revision, *INKA* inventory for complex attention, *IQ* Intelligence Quotient, *IVA CPT* integrated visual and auditory continuous performance test, *KBIT* Kaufman brief intelligence test, *N* number, *NA* not available, *NF* neurofeedback, *RT* reaction time, *VSWM* visual spatial working memory, *WISC* Wechsler intelligence scale for children, *SCF* standard computer format, *SCP* slow cortical potential training, *SST* Stop Signal test, *TAP* test for attentional performance, *TAU* treatment as usual, *TB* theta/beta training, *VCPT* visual continuous performance test, *WISC-III* Wechsler Intelligence Scale for Children, the 3rd edition, *WURS-K* short version of the German Wender Utah Rating Scale

^a^Studies that used protocols also including beta wave enhancement

A total of 718 participants with a mean age of 14.96 years (range, 8.66–37.8) and a female prevalence of 22.41% (range, 0–60.9%) were included. Among them, 429 received surface EEG-NF treatment. Of the 12 studies that used the TB ratio as their main EEG-NF treatment protocol, two also included beta wave enhancement in their protocols (Table 1). On the other hand, two studies used the SCP protocol [42, 43]. In the current meta-analysis, comparison groups consisted of TAU/waitlist and other non-pharmaceutical interventions (Table 1). Two studies used some forms of blinding such as sham control or sham electrode to blind their participants to treatment assignment [22, 38]. Of all the eligible studies, only three conducted a follow-up investigation. The diagnostic criteria for ADHD were mostly based on DSM-IV-TR with children being the main targeted group. Among the eligible studies, eight provided data on the intelligence quotient (IQ) of the testing subjects. Participants were not allowed to receive any stimulant medications in four out of the 14 studies, while all ADHD patients underwent medication treatment in one study by Lee and Jung [36].

Regarding the assessment of attentional performance, various tests were employed across the included studies, namely different versions of continuous performance test (CPT), stop-signal test, d2 test, Stroop task, working memory test, the subtests of Wechsler intelligence test, and attention network test. The number of EEG-NF sessions ranged from 20 to 40. Half of the studies (n = 7) were conducted in Germany, while two were performed in Asia [36, 45].

### Quantitative data synthesis

**Fig. 3:**
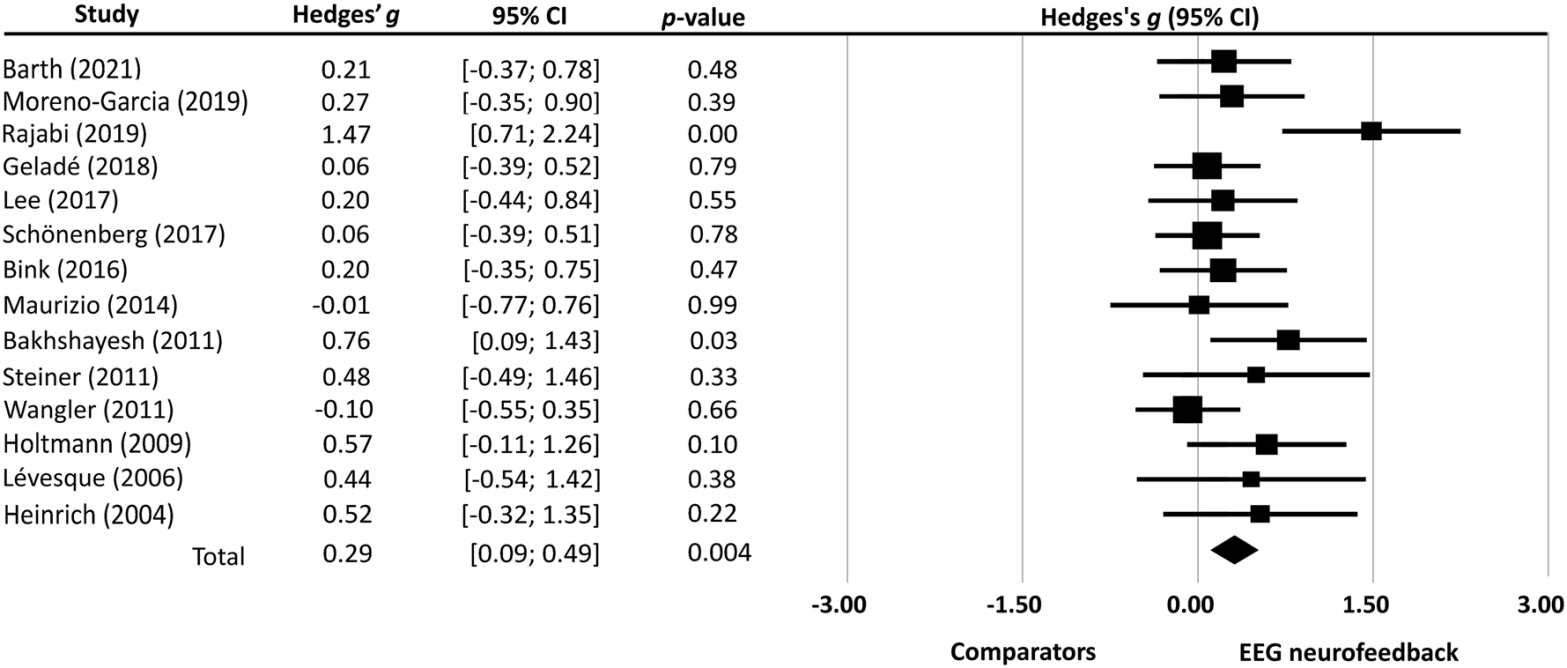
Forest plot of effect sizes for comparing the difference in the improvement of attentional performance between electroencephalographic (EEG) neurofeedback group and its comparators

The current meta-analysis of data from fourteen studies found more improvement in attentional performance in ADHD subjects receiving surface EEG-NF than that in their comparators (Hedges’ *g* = 0.29, *p* < 0.01) (Fig. 3). The assessment tools used in individual studies for different domains of attentional performance are provided in Additional file 4: Table S4. The ES was strong in the leave-one-out sensitivity analysis (*p* < 0.01), suggesting that the main result was not driven by any single study. Egger’s test was significant (*p* = 0.02), implicating a high risk of publication bias.

**Fig. 4:**
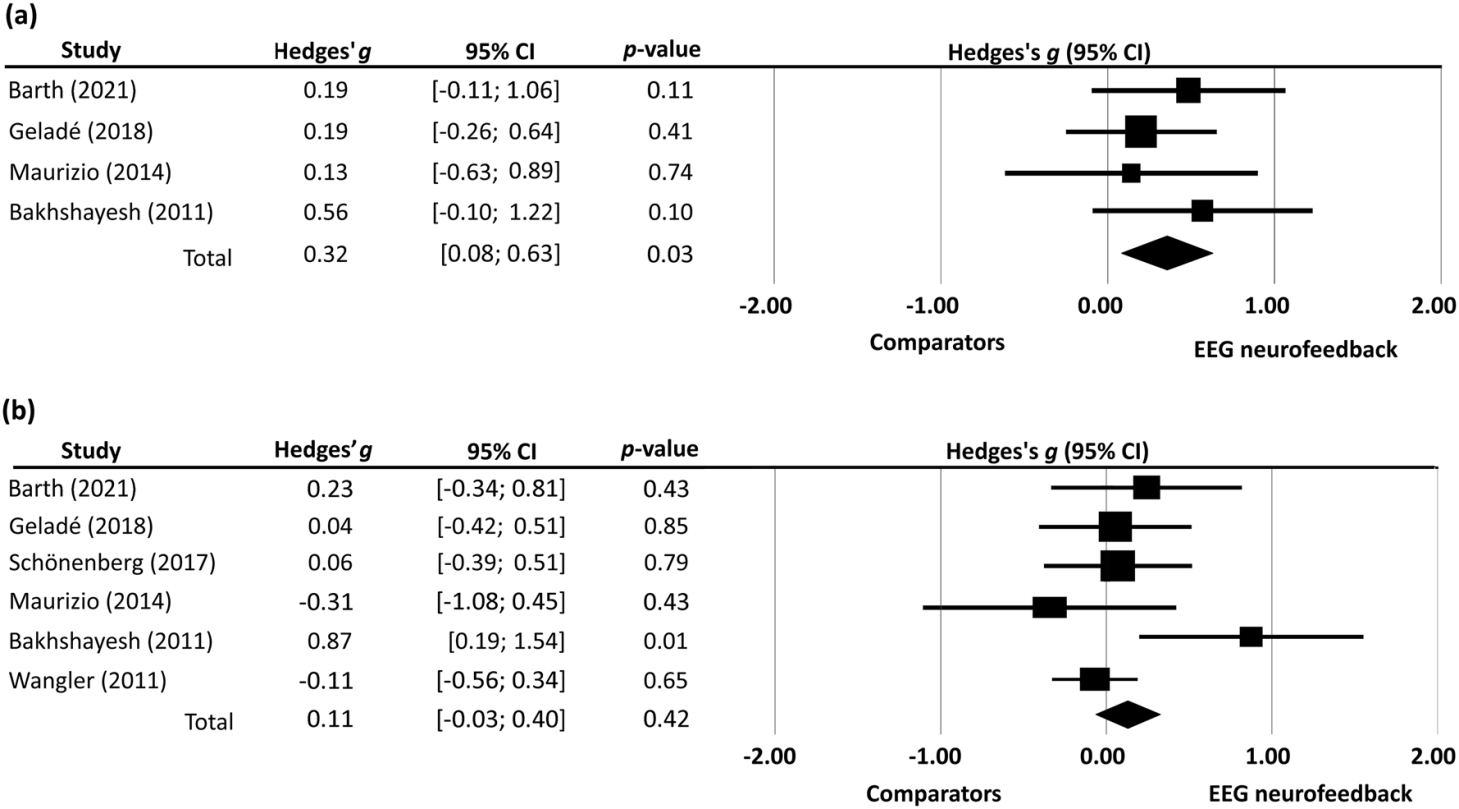
Forest plot of effect sizes for comparing the difference in **a** omissions and **b** reaction time between electroencephalographic (EEG) neurofeedback group and its comparators

Our results showed that EEG-NF had a significant beneficial impact on sustained attention (Hedges’ *g* = 0.32, *p* < 0.01), whereas its effects on selective attention (i.e., sustained attention plus cognitive control) [22, 34, 37, 38, 41, 44] (Hedges’ *g* = 0.07, *p* = 0.57) and working memory (e.g., digit span backwards) [34, 35] (Hedges’ *g* = 0.10, *p* = 0.59) were limited. Focusing on different components of sustained attention, there was a significant reduction in omissions (n = 4, Hedges’ *g* = 0.32, *p* = 0.03) but not in reaction time (n = 6, Hedges’ *g* = 0.11, *p* = 0.42) (Fig. 4a, b). Therefore, the findings suggested that surface EEG-NF could improve a subject’s sustained attention by reducing negligence.

Regarding the sustained effects of EEG-NF, only three studies provided follow-up data for attentional performance 6–12 months post-treatment [22, 34, 35]. A comparison of the results of follow-up assessments between EEG-NF and their comparators did not show a significant difference in the improvement of attentional performance 6 or 12 months after EEG-NF (*p* = 0.42).

### Subgroup analysis and meta-regression

Despite limited data availability, subgroup analyses of the impacts of the intensity of treatment, the quality of blinding, and the types of EEG-NF protocols on the improvement of attentional performance all showed a significant difference (all *p* < 0.05) (Table 2). In respect of the intensity of treatment, which was defined as the number of NF sessions per week, subgroup analysis revealed that three sessions per week was associated with a significantly higher ES than that with other intensities (i.e., one or two NF sessions per week) of EEG-NF intervention (*p* = 0.03). Besides, subgroup analysis showed significantly poorer therapeutic outcomes of surface EEG-NF in studies that involved participants who were unaware of treatment allocation compared to those in studies without blinding of treatment allocation to their participants (*p* = 0.006). Finally, the therapeutic efficacy of EEG-NF in studies that also focused on rewarding to beta activities was superior to that in those adopting only the TB ratio or the SCP protocol (*p* = 0.02) Using the mixed-effects model, our meta-regression demonstrated a significant negative correlation between the therapeutic benefits of surface EEG-NF and the percentage of females (*p* = 0.02). Otherwise, there was no significant association between the therapeutic benefits of surface EEG-NF and other continuous variables (Table 3).

**Table 2:** Subgroup analysis of factors affecting therapeutic benefits of electroencephalographic neurofeedback (EEG-NF) in subjects with attention deficit hyperactivity disorder (ADHD) and their comparators (Hedges’ *g*)

Subgroup analysis	N_comp_	*g*^a^	95% CI	Z	*Q*^b^	*P*^c^
Treatment intensity (i.e., number of sessions per week)						
One	3	0.24	− 0.12 to 0.61	1.30	1.93	0.03
Two	6	0.14	− 0.08 to 0.35	1.24		
Three or more	4	0.62	− 0.04 to 1.27	1.84		
Blinding to participants						
No	12	0.35	0.12 to 0.58	3.02^**^	1.76	0.006
Yes	2	0.05	− 0.34 to 0.43	0.23		
Types of neurofeedback						
Also including beta wave enhancement	2	0.82	− 0.43 to 2.06	1.28	1.13	0.02
Focusing on TB ratio	10	0.19	− 0.001 to 0.37	1.95		
Basing on SCPs	2	0.31	− 0.17 to 0.78	1.27		

^a^According to the random effects model

^b^Cochran’s *Q* for measuring the heterogeneity in accordance with random effects analysis

^c^Significance of difference in effect sizes between subgroups; N_comp_, Number of studies for comparison; TB, theta/beta; SCPs, Slow cortical potentials

^**^p < 0.01 between EEG-NF and control groups

**Table 3:** Regression coefficients of improvement in attentional performance in included studies using mixed-effects model

Continuous variable	N		Coefficient (95% CI)	*P*
Sex (percentage of female)	14		− 0.008 (− 0.01 to − 0.001)	0.02
Age	14		− 0.009 (− 0.028 to 0.01)	0.39
Treatment duration	14		0.006 (− 0.02 to 0.03)	0.67
Intelligent quotient	8		0.01 (− 0.05 to 0.08)	0.68
Percentage of medication	13		0.003 (− 0.002 to 0.009)	0.26
Percentage of inattention subtype	8		0.003 (− 0.01 to 0.02)	0.67
Percentage of combined subtype	6		− 0.005 (− 0.03 to 0.02)	0.66
Dropout rate	9		0.01 (− 0.03 to 0.05)	0.61

*N* number of studies, *CI* confidence interval

## Discussion

To our best knowledge, the present study is the first meta-analysis to investigate the efficacy of surface EEG-NF for improving different components of attentional performance in patients with ADHD assessed with objective laboratory measures. Although one previous meta-analysis reported that surface EEG-NF was not effective in ADHD patients when laboratory measures of attentional performance were used for the evaluation of treatment outcomes, that study included only six trials and did not address different components of attentional performance [7]. In contrast, the inclusion of more trials (n = 14) in the current meta-analysis enabled the assessment of the therapeutic impacts of EEG-NF on different components of attentional performance as well as the identification of important factors that could influence treatment outcomes through subgroup analysis and meta-regression.

Most of the previous seven meta-analyses of the efficacy of surface EEG-NF in patients with ADHD evaluated treatment outcomes mainly based on behavioral rating with the inclusion of laboratory measures only in one study [5, 7, 9–13]. All studies consistently found that surface EEG-NF was effective for improving inattention in ADHD patients when assessed by most-proximal evaluators, mostly parents, while the results from possibly blind evaluators (i.e., teachers) remained inconsistent [5, 7, 9–13]. The latest meta-analysis of 17 trials demonstrated that surface EEG-NF mainly improved inattention symptoms in ADHD patients when compared with the non-active control groups (i.e., waiting list) but the improvement was still more obvious when assessed by most-proximal evaluators (ES: − 0.33, i.e. parents) than that by possibly blind evaluators (ES: − 0.25, i.e. teacher) [5]. A previous study has also underscored the high susceptibility of the behavioral rating scales to informant bias [20], which could be even more significant when the intervention is not blinded to the participants. Moreover, the possibly blind evaluators may not be blinded because none of the trials actually prevented their participants from informing their evaluators (e.g., teachers) of the treatments that they received. This is partly supported by the only meta-analysis investigating laboratory measures of attentional performance that showed a further reduction in ES from 0.29 to 0.13 when laboratory measures were used for evaluating behavioral outcomes [7].

In contrast, laboratory measures of attentional performance may be less susceptible to informant bias [14] and could theoretically generate more reliable results. Besides, since improvement in neurocognitive functioning (i.e., endophenotype) should theoretically precede behavioral manifestation (i.e., phenotype), assessment of the former with attention tests in the laboratory setting may serve as an earlier indicator of EEG-NF-associated improvement than evaluation of the latter with behavioral rating scales [46]; therefore, laboratory measures are expected to more sensitively and accurately reflect the desired improvements in attentional performance from surface EEG-NF than behavioral changes from parents’ observations. Overall, we found a larger effect size regarding the improvement in attentional performance assessed by laboratory measures than that reported in a previous meta-analysis (Hedges’ *g* = 0.29 vs. 0.13, respectively) [7]. In contrast to behavioral rating scales, our meta-analysis provided important evidence from a different perspective.

Our subgroup analysis also demonstrated a significantly poorer therapeutic effect of surface EEG-NF on attentional performance in ADHD patients when participants were blinded to their treatment assignments (Hedges’ *g* = 0.05, *p* = 0.006). Apart from observation bias in favor of surface EEG-NF, a lack of blinding to participants may also lead to an enhancement of attentional performance due to increased motivation, which is commonly known as Hawthorne effect. Previous evidence showed that placebo effects, confidence in technology and therapeutic alliance along with other therapeutic factors could all contribute to therapeutic effects of EEG-NF through increasing motivation [47–49]. On the other hand, expectancy of self-efficacy, which is a crucial element of neurofeedback interventions [50], may be hampered in placebo-controlled intervention studies. Because only two studies blinded their participants to treatment allocation in our subgroup analysis, the finding regarding the effect of blinding of treatment allocation to participants on their improvement in attentional function needs to be interpreted with caution.

Our subgroup analysis focusing on different components of attentional performance further demonstrated a significant positive impact of surface EEG-NF only on sustained attention without significant effects on selective attention and working memory. Although motivation is important for both sustained attention and working memory [51, 52], the latter may require training involving multi-domains or more specific areas of the brain because it is generally accepted to be part of higher-order cognitive abilities [53]. Together with the finding that surface EEG-NF was considerably less effective when participant were blinded to treatment allocation and when laboratory measures for assessment were adopted, our results suggest that both motivation and confidence in surface EEG-NF may be important factors for the observed effectiveness and surface EEG-NF could only improve sustained attention which is considered a more basic attentional function [54].

Our subgroup analysis revealed that surface EEG-NF protocols including beta wave enhancement were more effective than those using SCP modulation or those focusing only on the TB ratio, indicating that the inclusion of beta wave enhancement was a better strategy. Evidence has shown that suppressed beta activities correlate with inattention and hyperactive symptoms, while increased theta activities are associated with impulsive behaviors [55]. However, the purely negative effect of theta activity on attention remains controversial as it is also associated with insight and creativity [6]. Therefore, the rationale for reducing the TB ratio to improve attentional performance may not be well justified. Additionally, our result was supported by another study that reported no significant difference in theta activities between children with ADHD and their normal developing counterparts, although ADHD children with a slow reaction time were found to have lower beta activities than those in normal developing children [19]. Therefore, consistent with the result of that study [19], our finding supports that enhancing beta activity may be a more effective target of EEG-NF than reducing the TB ratio for ADHD patients. Nevertheless, of the 14 studies included in the current meta-analysis, 12 investigated protocols addressing the TB ratio with only two also including beta wave enhancement. Similarly, although the ES of trials using the SCP protocol was slightly larger than that in those adopting the TB protocol, only two studies used the SCP protocol in our subgroup analysis. Despite the preliminary nature of the finding, our results may provide a direction for further investigations into the efficacy of different EEG-NF protocols for attentional training in patients with ADHD.

Our analysis on the sustained effects of EEG-NF at 6–12 months post-treatment failed to show significant difference in attentional performance between the EEG-NF and comparison groups, although this finding was derived from only three available trials [22, 34, 35]. In contrast, a previous meta-analysis demonstrated sustained treatment effects of surface EEG-NF for at least 6 months [9], while evidence was mixed from other reports [22, 34, 56, 57]. Because we could only include three trials for analyzing the long-term effects (i.e., over 6 months), our results need to be interpreted with caution. Nevertheless, given the potential concerns about the cost-effectiveness of EEG-NF [6], the sustainability of its therapeutic benefits and the need for boosting sessions in the long run remain important questions to guide clinical decision for both clinicians and caregivers (e.g., patients), further studies are warranted to address this issue.

In the present study, subgroup analysis focusing on different intensities of treatment suggested that participants with NF training for at least three sessions per week achieved significantly better improvement in sustained attention than those with a lower treatment intensity. A shorter interval between training sessions may be associated with a more significant increase in synaptic efficacy according to the concept of neuroplasticity in a process called Hebbian plasticity [58]. Although the ES of EEG-NF with one session per week was higher than that in programs offering two sessions per week, inclusion of one large-scale study choosing the intensity of two sessions per week with a sham condition [22] may lead to a lower efficacy of EEG-NF. In contrast, the ES of trials with three sessions per week remained the largest despite the inclusion of one study using sham electrodes [38], suggesting that EEG-NF with more frequent training may achieve better therapeutic outcomes.

In addition, our meta-regression analysis found that female gender correlated with poorer therapeutic effects of EEG-NF. This result needs to be judiciously interpreted as most trials enrolled far more males than females. In particular, of the two trials that did not recruit female participants, one focused on beta enhancement and showed a very large effect size [45]. Therefore, the choice of treatment protocol, rather than gender, may contribute to the difference in treatment efficacy. Consistently, a previous trial including a more balanced gender profile failed to identify a significant influence of gender on neurofeedback learning [59]. The current study demonstrated that other demographic factors, medication use, and subtypes of ADHD were not associated with the efficacy of surface EEG-NF.

Despite the strength of the current study as the first meta-analysis to explore the therapeutic impact of surface EEG-NF on different components of attentional performance in patients with ADHD evaluated with objective laboratory measures, there were several limitations that need to be considered for accurate interpretations of its findings. First, although we were able to include more trials compared with a previous meta-analysis [7], the number of trials (14 RCTs) and participants (n = 718) were still too small to provide tangible evidence. In particular, our preliminary finding on the long-term therapeutic effects of EEG-NF, which was derived from only three trials that provided information about outcomes at 6–12 months follow-ups [22, 34, 35], could not preclude the potential benefits of prolonged EEG-NF treatment. Second, most studies had poor methodological qualities regarding performance bias and detection bias. However, our study used more objective measures of attentional performance in a laboratory-based setting, which is another major strength of this meta-analysis. Third, the heterogeneity of the included studies, including differences in the types of EEG-NF protocols, the control conditions from no active treatment to medications, and the recruited age groups, may limit generalization of our results. Nevertheless, we performed subgroup analysis and meta-regression to address those specific issues. Fourth, our findings only supported significant improvements in attention outcomes associated with surface EEG-NF intervention based on continuous variables, but were unable to provide clear information regarding the clinical significance when using parameters such as number needed to treat (NNT). Finally, taking into account the significant publication bias shown in the present meta-analysis and the lack of blinding in most studies as well as the very small effect size, the true effect of surface EEG NF on attentional performance needs to be interpreted with the utmost discretion.

## Conclusion

In general, focusing on laboratory measures, our results supported the use of surface EEG-NF for improving attentional performance through the modulation of basic neurocognitive functioning in patients with ADHD. The current study suggested that surface EEG-NF with beta wave enhancement may be a more effective protocol for improving sustained attention through reducing omissions but this effect failed to sustain a follow-up period longer than 6 months. However, given the small number of trials and the poor methodological qualities regarding blinding, our findings need to be judiciously interpreted and warrant further investigations for validation.

## Supplementary Information

**Additional file 1: Table S1.** Applied keyword and the search result in each database.

**Additional file 2: Table S2.** PRISMA checklist of the present meta-analysis.

**Additional file 3: Table S3.** Reasons for study exclusion.

**Additional file 4:Table S4.** Components of attention tests targeting different cognitive domains in the included studies.

## Data Availability

The datasets used and/or analyzed during the current study are available from the corresponding author on reasonable request.

## Abbreviations

ADHD: Attention-deficit/hyperactivity disorder
CMA: Comprehensive Meta-Analysis version
CPT: Continuous performance test
DSM-IV-TR: The Diagnostic and Statistical Manual of Mental Disorders, fourth edition, text revision
EEG-NF: Electroencephalographic neurofeedback
ES: Effect size
fMRI: Functional magnetic resonance imaging
Fz: Frontal
IQ: Intelligent quotient
LC: Locus coeruleus
NNT: Number needed to treat
RCT: Randomized control trial
SCP: Slow cortical potential
TAU: Treatment as usual
TB: Theta/beta
