# Supplementary material for: Surface electroencephalographic neurofeedback improves sustained attention in ADHD: a meta-analysis of randomized controlled trials
Source: Child Adolesc Psychiatry Ment Health. 2022 Dec 19;16:104. doi: 10.1186/s13034-022-00543-1 (PMC9764556; doi:10.1186/s13034-022-00543-1)
Supplement: Supplementary file 1 — Additional file 1: Table S1. Applied keyword and the search result in each database. [file 13034_2022_543_MOESM1_ESM.docx]

**Table S1** Applied keywords and the search results in each database

| Database | Keywords | Filter | Date | Results |
| --- | --- | --- | --- | --- |
| PubMed | (neurofeedback) AND (attention or attention-deficit/hyperactivity disorder or ADHD) | RCT | 2022/01/01 | 95 |
| Embase | (neurofeedback) AND (attention or ADHD) | RCT | 2022/01/01 | 156 |
| ClinicalKey | (neurofeedback) AND (attention or attention-deficit/hyperactivity disorder or ADHD) | RCT | 2022/01/01 | 56 |
| Cochrane CENTRAL | (neurofeedback) AND (attention or attention-deficit/hyperactivity disorder or ADHD) | Trials | 2022/01/01 | 343 |
| ScienceDirect | (neurofeedback) AND (attention or attention-deficit/hyperactivity disorder or ADHD) | Research article | 2022/01/01 | 201 |
| Web of Science | (neurofeedback) AND (attention or attention deficit hyperactivity disorder or ADHD) | Article | 2022/01/01 | 678 |
| ClinicalTrials.gov | (ADHD) AND (neurofeedback) | NA | 2021/08/31 | 27 |

Abbreviations: NA, not applied; RCT, randomized controlled trial
