# Supplementary material for: Surface electroencephalographic neurofeedback improves sustained attention in ADHD: a meta-analysis of randomized controlled trials
Source: Child Adolesc Psychiatry Ment Health. 2022 Dec 19;16:104. doi: 10.1186/s13034-022-00543-1 (PMC9764556; doi:10.1186/s13034-022-00543-1)
Supplement: Supplementary file 3 — Additional file 3: Table S3. Reasons for study exclusion. [file 13034_2022_543_MOESM3_ESM.docx]

**Table S3** Reasons for study exclusion

| **Reason** | **Number of excluded studies** | | | **References** |  |
| --- | --- | --- | --- | --- | --- |
| No outcome for attention test | | 17 | (Aggensteiner et al., 2019, Lim et al., 2019, Minder et al., 2018, Duric et al., 2017, Sudnawa et al., 2018, Strehl et al., 2017, Bink et al., 2015, Li et al., 2013, Maurizio et al., 2014, Steiner et al., 2014a, Meisel et al., 2014, Christiansen et al., 2014, Arnold et al., 2013, Lansbergen et al., 2011, Gevensleben et al., 2010, Neurofeedback Collaborative Group, 2021, van Dongen-Boomsma et al., 2013) | | |
| Not RCT | | 9 | (Holtmann et al., 2014, Riesco-Matías et al., 2021, Van Doren et al., 2019, Bioulac et al., 2019, Rossiter, 2004, Fuchs et al., 2003, Ryoo and Son, 2015, Qian et al., 2018, Pakdaman et al., 2018) | | |
| Duplicated sample source | | 7 | (Steiner et al., 2014b, Gevensleben et al., 2009b, Gevensleben et al., 2009a, Janssen et al., 2016, Meisel et al., 2013, Baumeister et al., 2019, Beauregard and Lévesque, 2006) | | |
| No available data for analysis | | 3 | (Norouzi et al., 2018, Logemann et al., 2010, Baumeister et al., 2018) | | |
| Not EEG neurofeedback | | 5 | (Rubia et al., 2019, Zilverstand et al., 2017, Criaud et al., 2020, Sherwood et al., 2019, Lam et al., 2020) | | |
| Only pharmacological interventions as control group | | 2 | (Ogrim and Hestad, 2013, Purper-Ouakil et al., 2021) | | |

RCT, randomized controlled trials; EEG, electroencephalogram

**References**

Aggensteiner, P. M., Brandeis, D., Millenet, S., Hohmann, S., Ruckes, C., Beuth, S., Albrecht, B., Schmitt, G., Schermuly, S., Wörz, S., Gevensleben, H., Freitag, C. M., Banaschewski, T., Rothenberger, A., Strehl, U. & Holtmann, M. (2019). Slow cortical potentials neurofeedback in children with ADHD: comorbidity, self-regulation and clinical outcomes 6 months after treatment in a multicenter randomized controlled trial. *Eur Child Adolesc Psychiatry,* 28**,** 1087-1095.

Arnold, L. E., Lofthouse, N., Hersch, S., Pan, X., Hurt, E., Bates, B., Kassouf, K., Moone, S. & Grantier, C. (2013). EEG neurofeedback for ADHD: double-blind sham-controlled randomized pilot feasibility trial. *J Atten Disord,* 17**,** 410-419.

Baumeister, S., Wolf, I., Hohmann, S., Holz, N., Boecker-Schlier, R., Banaschewski, T. & Brandeis, D. (2019). The impact of successful learning of self-regulation on reward processing in children with ADHD using fMRI. *Atten Defic Hyperact Disord,* 11**,** 31-45.

Baumeister, S., Wolf, I., Holz, N., Boecker-Schlier, R., Adamo, N., Holtmann, M., Ruf, M., Banaschewski, T., Hohmann, S. & Brandeis, D. (2018). Neurofeedback Training Effects on Inhibitory Brain Activation in ADHD: A Matter of Learning? *Neuroscience,* 378**,** 89-99.

Beauregard, M. & Lévesque, J. (2006). Functional magnetic resonance imaging investigation of the effects of neurofeedback training on the neural bases of selective attention and response inhibition in children with attention-deficit/hyperactivity disorder. *Appl Psychophysiol Biofeedback,* 31**,** 3-20.

Bink, M., van Nieuwenhuizen, C., Popma, A., Bongers, I. L. & van Boxtel, G. J. (2015). Behavioral effects of neurofeedback in adolescents with ADHD: a randomized controlled trial. *Eur Child Adolesc Psychiatry,* 24**,** 1035-1048.

Bioulac, S., Purper-Ouakil, D., Ros, T., Blasco-Fontecilla, H., Prats, M., Mayaud, L. & Brandeis, D. (2019). Personalized at-home neurofeedback compared with long-acting methylphenidate in an european non-inferiority randomized trial in children with ADHD. *BMC Psychiatry,* 19**,** 237.

Christiansen, H., Reh, V., Schmidt, M. H. & Rief, W. (2014). Slow cortical potential neurofeedback and self-management training in outpatient care for children with ADHD: study protocol and first preliminary results of a randomized controlled trial. *Front Hum Neurosci,* 8**,** 943.

Criaud, M., Wulff, M., Alegria, A. A., Barker, G. J., Giampietro, V. & Rubia, K. (2020). Increased left inferior fronto-striatal activation during error monitoring after fMRI neurofeedback of right inferior frontal cortex in adolescents with attention deficit hyperactivity disorder. *Neuroimage Clin,* 27**,** 102311.

Duric, N. S., Assmus, J., Gundersen, D., Duric Golos, A. & Elgen, I. B. (2017). Multimodal treatment in children and adolescents with attention-deficit/hyperactivity disorder: a 6-month follow-up. *Nord J Psychiatry,* 71**,** 386-394.

Fuchs, T., Birbaumer, N., Lutzenberger, W., Gruzelier, J. H. & Kaiser, J. (2003). Neurofeedback treatment for attention-deficit/hyperactivity disorder in children: a comparison with methylphenidate. *Appl Psychophysiol Biofeedback,* 28**,** 1-12.

Gevensleben, H., Holl, B., Albrecht, B., Schlamp, D., Kratz, O., Studer, P., Rothenberger, A., Moll, G. H. & Heinrich, H. (2010). Neurofeedback training in children with ADHD: 6-month follow-up of a randomised controlled trial. *Eur Child Adolesc Psychiatry,* 19**,** 715-724.

Gevensleben, H., Holl, B., Albrecht, B., Schlamp, D., Kratz, O., Studer, P., Wangler, S., Rothenberger, A., Moll, G. H. & Heinrich, H. (2009a). Distinct EEG effects related to neurofeedback training in children with ADHD: a randomized controlled trial. *Int J Psychophysiol,* 74**,** 149-157.

Gevensleben, H., Holl, B., Albrecht, B., Vogel, C., Schlamp, D., Kratz, O., Studer, P., Rothenberger, A., Moll, G. H. & Heinrich, H. (2009b). Is neurofeedback an efficacious treatment for ADHD? A randomised controlled clinical trial. *J Child Psychol Psychiatry,* 50**,** 780-789.

Holtmann, M., Pniewski, B., Wachtlin, D., Wörz, S. & Strehl, U. (2014). Neurofeedback in children with attention-deficit/hyperactivity disorder (ADHD)--a controlled multicenter study of a non-pharmacological treatment approach. *BMC Pediatr,* 14**,** 202.

Janssen, T. W., Bink, M., Geladé, K., van Mourik, R., Maras, A. & Oosterlaan, J. (2016). A randomized controlled trial into the effects of neurofeedback, methylphenidate, and physical activity on EEG power spectra in children with ADHD. *J Child Psychol Psychiatry,* 57**,** 633-644.

Lam, S. L., Criaud, M., Alegria, A., Barker, G. J., Giampietro, V. & Rubia, K. (2020). Neurofunctional and behavioural measures associated with fMRI-neurofeedback learning in adolescents with Attention-Deficit/Hyperactivity Disorder. *Neuroimage Clin,* 27**,** 102291.

Lansbergen, M. M., van Dongen-Boomsma, M., Buitelaar, J. K. & Slaats-Willemse, D. (2011). ADHD and EEG-neurofeedback: a double-blind randomized placebo-controlled feasibility study. *J Neural Transm (Vienna),* 118**,** 275-284.

Li, L., Yang, L., Zhuo, C. J. & Wang, Y. F. (2013). A randomised controlled trial of combined EEG feedback and methylphenidate therapy for the treatment of ADHD. *Swiss Med Wkly,* 143**,** w13838.

Lim, C. G., Poh, X. W. W., Fung, S. S. D., Guan, C., Bautista, D., Cheung, Y. B., Zhang, H., Yeo, S. N., Krishnan, R. & Lee, T. S. (2019). A randomized controlled trial of a brain-computer interface based attention training program for ADHD. *PLoS One,* 14**,** e0216225.

Logemann, H. N., Lansbergen, M. M., Van Os, T. W., Böcker, K. B. & Kenemans, J. L. (2010). The effectiveness of EEG-feedback on attention, impulsivity and EEG: a sham feedback controlled study. *Neurosci Lett,* 479**,** 49-53.

Maurizio, S., Liechti, M. D., Heinrich, H., Jäncke, L., Steinhausen, H. C., Walitza, S., Brandeis, D. & Drechsler, R. (2014). Comparing tomographic EEG neurofeedback and EMG biofeedback in children with attention-deficit/hyperactivity disorder. *Biol Psychol,* 95**,** 31-44.

Meisel, V., Servera, M., Garcia-Banda, G., Cardo, E. & Moreno, I. (2013). Neurofeedback and standard pharmacological intervention in ADHD: a randomized controlled trial with six-month follow-up. *Biol Psychol,* 94**,** 12-21.

Meisel, V., Servera, M., Garcia-Banda, G., Cardo, E. & Moreno, I. (2014). Reprint of "Neurofeedback and standard pharmacological intervention in ADHD: a randomized controlled trial with six-month follow-up". *Biol Psychol,* 95**,** 116-125.

Minder, F., Zuberer, A., Brandeis, D. & Drechsler, R. (2018). Informant-related effects of neurofeedback and cognitive training in children with ADHD including a waiting control phase: a randomized-controlled trial. *Eur Child Adolesc Psychiatry,* 27**,** 1055-1066.

Neurofeedback Collaborative Group (2020). Double-Blind Placebo-Controlled Randomized Clinical Trial of Neurofeedback for Attention-Deficit/Hyperactivity Disorder With 13-Month Follow-up. *J Am Acad Child Adolesc Psychiatry,* 60, 841-855.

Norouzi, E., Hossieni, F. & Solymani, M. (2018). Effects of Neurofeedback Training on Performing Bimanual Coordination In-phase and Anti-phase Patterns in Children with ADHD. *Appl Psychophysiol Biofeedback,* 43**,** 283-292.

Ogrim, G. & Hestad, K. A. (2013). Effects of neurofeedback versus stimulant medication in attention-deficit/hyperactivity disorder: a randomized pilot study. *J Child Adolesc Psychopharmacol,* 23**,** 448-457.

Pakdaman, F., Irani, F., Tajikzadeh, F. & Jabalkandi, S. A. (2018). The efficacy of Ritalin in ADHD children under neurofeedback training. *Neurol Sci,* 39**,** 2071-2078.

Purper-Ouakil, D., Blasco-Fontecilla, H., Ros, T., Acquaviva, E., Banaschewski, T., Baumeister, S., Bousquet, E., Bussalb, A., Delhaye, M., Delorme, R., Drechsler, R., Goujon, A., Häge, A., Kaiser, A., Mayaud, L., Mechler, K., Menache, C., Revol, O., Tagwerker, F., Walitza, S., Werling, A. M., Bioulac, S. & Brandeis, D. (2021). Personalized at-home neurofeedback compared to long-acting methylphenidate in children with ADHD: NEWROFEED, a European randomized noninferiority trial. *J Child Psychol Psychiatry*.

Qian, X., Loo, B. R. Y., Castellanos, F. X., Liu, S., Koh, H. L., Poh, X. W. W., Krishnan, R., Fung, D., Chee, M. W., Guan, C., Lee, T. S., Lim, C. G. & Zhou, J. (2018). Brain-computer-interface-based intervention re-normalizes brain functional network topology in children with attention deficit/hyperactivity disorder. *Transl Psychiatry,* 8**,** 149.

Riesco-Matías, P., Yela-Bernabé, J. R., Crego, A. & Sánchez-Zaballos, E. (2021). What Do Meta-Analyses Have to Say About the Efficacy of Neurofeedback Applied to Children With ADHD? Review of Previous Meta-Analyses and a New Meta-Analysis. *J Atten Disord,* 25**,** 473-485.

Rossiter, T. (2004). The effectiveness of neurofeedback and stimulant drugs in treating AD/HD: part II. Replication. *Appl Psychophysiol Biofeedback,* 29**,** 233-243.

Rubia, K., Criaud, M., Wulff, M., Alegria, A., Brinson, H., Barker, G., Stahl, D. & Giampietro, V. (2019). Functional connectivity changes associated with fMRI neurofeedback of right inferior frontal cortex in adolescents with ADHD. *Neuroimage,* 188**,** 43-58.

Ryoo, M. & Son, C. (2015). Effects of Neurofeekback Training on EEG, Continuous Performance Task (CPT), and ADHD Symptoms in ADHD-prone College Students. *J Korean Acad Nurs,* 45**,** 928-938.

Sherwood, M. S., Parker, J. G., Diller, E. E., Ganapathy, S., Bennett, K. B., Esquivel, C. R. & Nelson, J. T. (2019). Self-directed down-regulation of auditory cortex activity mediated by real-time fMRI neurofeedback augments attentional processes, resting cerebral perfusion, and auditory activation. *Neuroimage,* 195**,** 475-489.

Steiner, N. J., Frenette, E. C., Rene, K. M., Brennan, R. T. & Perrin, E. C. (2014a). In-school neurofeedback training for ADHD: sustained improvements from a randomized control trial. *Pediatrics,* 133**,** 483-492.

Steiner, N. J., Frenette, E. C., Rene, K. M., Brennan, R. T. & Perrin, E. C. (2014b). Neurofeedback and cognitive attention training for children with attention-deficit hyperactivity disorder in schools. *J Dev Behav Pediatr,* 35**,** 18-27.

Strehl, U., Aggensteiner, P., Wachtlin, D., Brandeis, D., Albrecht, B., Arana, M., Bach, C., Banaschewski, T., Bogen, T., Flaig-Röhr, A., Freitag, C. M., Fuchsenberger, Y., Gest, S., Gevensleben, H., Herde, L., Hohmann, S., Legenbauer, T., Marx, A. M., Millenet, S., Pniewski, B., Rothenberger, A., Ruckes, C., Wörz, S. & Holtmann, M. (2017). Neurofeedback of Slow Cortical Potentials in Children with Attention-Deficit/Hyperactivity Disorder: A Multicenter Randomized Trial Controlling for Unspecific Effects. *Front Hum Neurosci,* 11**,** 135.

Sudnawa, K. K., Chirdkiatgumchai, V., Ruangdaraganon, N., Khongkhatithum, C., Udomsubpayakul, U., Jirayucharoensak, S. & Israsena, P. (2018). Effectiveness of neurofeedback versus medication for attention-deficit/hyperactivity disorder. *Pediatr Int,* 60**,** 828-834.

van Dongen-Boomsma, M., Vollebregt, M. A., Slaats-Willemse, D. & Buitelaar, J. K. (2013). A randomized placebo-controlled trial of electroencephalographic (EEG) neurofeedback in children with attention-deficit/hyperactivity disorder. *J Clin Psychiatry,* 74**,** 821-827.

Van Doren, J., Arns, M., Heinrich, H., Vollebregt, M. A., Strehl, U. & S, K. L. (2019). Sustained effects of neurofeedback in ADHD: a systematic review and meta-analysis. *Eur Child Adolesc Psychiatry,* 28**,** 293-305.

Zilverstand, A., Sorger, B., Slaats-Willemse, D., Kan, C. C., Goebel, R. & Buitelaar, J. K. (2017). fMRI Neurofeedback Training for Increasing Anterior Cingulate Cortex Activation in Adult Attention Deficit Hyperactivity Disorder. An Exploratory Randomized, Single-Blinded Study. *PLoS One,* 12**,** e0170795.
