# Supplementary material for: Surface electroencephalographic neurofeedback improves sustained attention in ADHD: a meta-analysis of randomized controlled trials
Source: Child Adolesc Psychiatry Ment Health. 2022 Dec 19;16:104. doi: 10.1186/s13034-022-00543-1 (PMC9764556; doi:10.1186/s13034-022-00543-1)
Supplement: Supplementary file 4 — Additional file 4:Table S4. Components of attention tests targeting different cognitive domains in the included studies. [file 13034_2022_543_MOESM4_ESM.docx]

Table S4: Components of attention tests targeting different cognitive domains in the included studies

|  | **Sustained attention**  **(test**：**components)** | **Selective attention**  **(test**：**components)** | **Working memory**  **(test**：**components)** |
| --- | --- | --- | --- |
| Barth (2021) | Go/No Go task：  RT + omission + errors | D2 Test：accuracy + concentration + speed |  |
| Moreno-García (2019) | IVA CPT：full Scale |  |  |
| Rajabi (2019) | IVA CPT：full Scale |  |  |
| Geladé (2018) | 1. Auditory oddball task：RT+ CV 2. SST：RT + omission |  | SWM：backward |
| Lee (2017)* | 1. ADS：inattention  2. Korean WISC-III：freedom from distractibility |  |  |
| Schönenberg  (2017) | 1. INKA 2. CPT：RT | Stroop word and color test：total score |  |
| Bink (2016) |  | 1. D2 Test：total score  2. Stroop word and color test：total score | Digit span：backward |
| Maurizio  (2014) | 1. KiTAP：RT + errors + hits+ omission 2. TAP：RT | D2 Test：total score |  |
| Bakhshayesh (2011) | CPT：total score + omission + RT |  |  |
| Steiner (2011) | IVA CPT：attention quotient |  |  |
| Wangler (2011) | ANT：alerting + conflict + orienting + hit + RT |  |  |
| Holtmann (2009) | SST：inattention |  |  |
| Lévesque (2006) | IVA CPT：attention quotient | Stroop task：inference |  |
| Heinrich (2004) | CPT：Hits |  |  |

Abbreviations: ADS, ADHD diagnostic system; ANT, Attention Network Test; CPT, continuous performance task; CV, coefficient of variation; D2 test, test of attention; KiTAP, test for attentional performance for children; INKA, inventory for complex attention; IVA CPT, integrated visual and auditory continuous performance test; RT, reaction time; VSWM, visual spatial working memory, WISC, Wechsler intelligence scale for children; SST, Stop Signal test; TAP, test for attentional performance; VCPT, visual continuous performance test; WISC-III, Wechsler Intelligence Scale for Children, the 3rd edition
